# The efficacy of autologous serum in corneal healing following metallic foreign body injuries

**DOI:** 10.1007/s00417-025-06866-x

**Published:** 2025-06-11

**Authors:** Levent Doğan, Ömer Özer, Zeki Baysal, Özer Dursun

**Affiliations:** 1 Department of Ophthalmology, Niğde Ömer Halisdemir University, Niğde, 51240 Turkey; 2 Department of Ophthalmology, Mersin University, Mersin, Turkey

**Keywords:** Metallic corneal foreign body, Autologous serum, Epithelial defect, Epithelial healing rate, Corneal densitometry

## Abstract

**Purpose:**

To evaluate the effect of topical autologous serum (AS) on corneal healing following metallic corneal foreign body (CFB) trauma.

**Method:**

Following the removal of CFBs with a 26-gauge needle, patients were randomly divided into two groups. Group 1 patients received topical moxifloxacin and preservative- free artificial tears, while Group 2 participants used these drops in addition to AS five times a day. Patients were followed until their corneal epithelial defects were closed. The baseline size of the epithelial defects was measured, and corneal densitometry measurements using Pentacam HR (Oculus, Wetzlar, Germany) were obtained before treatment. Epithelial healing rates and post-treatment corneal densitometric measurements were compared.

**Results:**

Group 1 comprised 41 male participants with a mean age of 36.1 ± 13.3 years, and Group 2 consisted of 42 male participants with a mean age of 34.9 ± 11.5 years. No statistically significant difference in age distribution was observed between the groups (*p* = 0.229). Complete epithelial closure occurred within 3.51 ± 1.03 days for Group 1 participants, whereas Group 2 participants achieved complete closure within 2.19 ± 1.10 days. A significantly faster rate of epithelial closure and a notably shorter duration were observed in Group 2 (*p* = 0.002 and *p* < 0.001, sincerely). Post-epithelial healing corneal densitometry measurements revealed significantly lower values in participants utilizing AS (*p* = 0.007).

**Conclusion:**

The use of AS accelerated corneal defect healing following metallic CFB removal and resulted in a greater reduction in corneal densitometry values compared with standard treatment with topical antibiotics and artificial tears.

## Key messages

*****What is known***:**

- Corneal metallic foreign bodies are one of the most common reasons for visits to ophthalmic emergencies and, if not properly healed, can lead to permanent visual impairments.
- Topical autologous serum can enhance the healing of corneal persistent epithelial defects.

*****What is new***:**

- The use of autologous serum accelerates corneal defect healing following metallic CFB removal.
- The use of autologous serum can decrease the corneal densitometry measurements compared with conventional treatments in corneal foreign body injuries.

## Introduction

Superficial corneal foreign bodies (CFBs) are a common reason for emergency department presentations, with corneal metallic foreign bodies constituting a significant proportion of these cases [1]. Clinical manifestations of metallic CFB injuries include pain, increased lacrimation, photophobia, conjunctival hyperemia, and decreased visual acuity [1, 2]. They can even lead to complications such as infectious and inflammatory keratitis, which may have a direct and adverse effect on visual prognosis [2]. Timely and appropriate debridement of corneal foreign bodies is crucial for optimal visual recovery [3]. To mitigate the risk of post-excision infection and facilitate a more comfortable epithelial healing process, topical antibiotics and artificial tears are recommended [4].

Autologous serum (AS), like natural tears, contains epidermal growth factor (EGF), transforming growth factor-beta (TGF-β), and vitamin A, exerting an epitheliotropic effect on the ocular surface [5]. The initial publication on the use of AS in ophthalmology dates to 1975, when Ralph et al. applied artificial tears, autologous and allogeneic serum, and plasma drops to the ocular surface using an ocular perfusion pump device [6]. This novel approach was employed to treat a patient with an alkali burn. Since then, AS eye drops have been refined and are now widely used for various ocular indications, including superior limbic keratoconjunctivitis, severe dry eye syndrome, persistent corneal epithelial defects, chemical burns, and neurotrophic keratitis [7–9].

The presence of epithelial defects is a significant risk factor for the development of microbial keratitis [10]. Accelerating the healing of these defects in patients with CFBs not only reduces the risk of keratitis but also improves patient comfort and facilitates a quicker return to social and occupational activities. A comprehensive literature search revealed no previous studies investigating the efficacy of AS in the treatment of CFB injuries. Consequently, this study aimed to evaluate the impact of topical AS applications on the healing process following CFB removal.

## Method

This prospective study was approved by the local ethics committee (approval number: 2024/2) and conducted in accordance with the Declaration of Helsinki. Following a detailed explanation of the study protocol, procedures, and planned measurements, written and verbal informed consent was obtained from all participants. The study recruited participants from patients who were consecutively admitted to the ophthalmology emergency department for CFB injuries caused by metallic foreign bodies between February and August 2024.

Following the topical administration of proparacaine to the patients’ injured eyes, CFBs and dust rings were removed using a 26-gauge needle on the biomicroscope by an experienced ophthalmologist (LD). Following CFBs removal, the epithelial defect was assessed using slit-lamp biomicroscopy under cobalt blue light with fluorescein. Corneal photographs were captured. The epithelial defect size was measured using ImageJ software (National Institutes of Health, Bethesda, Maryland, USA) [11]. Corneal defect depth was evaluated using Pentacam HR (Oculus, Wetzlar, Germany) and graded as involving the epithelium-basement membrane, anterior stroma, or mid-stroma. Corneal densitometry was conducted using the Pentacam, following a previously described protocol; a series of 25 images (1003 × 520 pixels) were captured across multiple meridians using a uniform blue light source [12]. The device automatically identified the corneal apex and analyzed a 12-mm diameter region, quantifying light scatter in grayscale units (GSU). The 12-mm diameter region was further subdivided into four concentric zones: a central 2-mm zone, a second annulus (2–6 mm), a third annulus (6–10 mm), and a peripheral zone (10–12 mm). Additionally, corneal densitometry was calculated for anterior and central layers. The mean density of the areas corresponding to the corneal surface annulus (central, second, third, or peripheral) and the depth of the defect secondary to CFB removal were recorded as the final density. Measurements were evaluated by two ophthalmologists (ÖÖ, ZB), and the average of their assessments was used.

A detailed eye examination was performed on all participants in the first consultation, including uncorrected distance visual acuity (UCDVA) (logMAR) measurement, anterior segment examination using slit-lamp biomicroscopy, dilated fundoscopic examination, tear breakup-time measurement (TBUT), and Schirmer I test with topical anesthesia after the CFB removal. In TBUT assessment, the first tear film break-up time in areas not adjacent to the epithelial defect was calculated and evaluated. CFB type and size, affected corneal area, and presence of anterior chamber response were recorded.

Patients were randomized into two groups, and the ophthalmologists performing the measurements were blinded to the patients’ treatment regimens. Group 1 received topical moxifloxacin and preservative- free, 0.15% sodium hyaluronate artificial tears five times daily, while Group 2 received the same medications in addition to AS eye drops five times daily. We advised the patients to instill moxifloxacin first, followed by artificial tears after a 10-minute interval, and subsequently, for patients in Group 2, to instill AS after an additional 10-minute interval for each administration. Prior to AS use, all patients underwent serological testing for syphilis, hepatitis B, C, and HIV. Twenty milliliters of blood was collected from each patient, allowed to clot at room temperature for two hours, and then centrifuged at 1500 rpm for 10 min. The supernatant serum was transferred to a sterile eye drop bottle and diluted 1:4 with sterile saline to achieve a 20% serum concentration. All patients scheduled to use autologous serum eye drops were instructed to store the eye drop bottle in a refrigerator (4 °C), transport it in a cooling container when removed, and protect it from ultraviolet light. Using the G Power program, the number of samples to be included in this study was determined as 19 with type I error (α = 5%), confidence (1-α = 95%), type II error (β = 10%), test power (1-β = 90%), and effect size (d = 0.5).

Following the initiation of medical therapy, patients were evaluated daily to monitor epithelialization. The effectiveness of treatment in promoting epithelialization, the rate of epithelialization (calculated by dividing the initial epithelial defect area by the number of days to complete epithelialization in mm²/day), and the time to complete healing were assessed. Complete healing was defined as the absence of fluorescein staining on the cornea. Once the defect was observed to have healed, the tear TBUT, Schirmer’s test, and corneal densitometry measurements in the areas of the initial defect were repeated and noted. Eyes with CFBs that did not penetrate beyond the mid-stroma were included in the study. Patients were excluded from the study if they missed follow-up visits during the epithelialization process, presented more than two days post-trauma, exhibited keratitis or anterior segment reaction, had a diagnosis of diabetes mellitus, immunodeficiency, pre-existing corneal pathology or opacity, or a history of dry eye disease or treatment. A comparative analysis was conducted between the data obtained from patients who used AS and those who did not.

### Statistical analysis

Statistical analysis was performed using SPSS, version 26.0, for Windows (IBM Corporation, Armonk, NY). Descriptive statistics are presented as numbers and percentages for categorical variables and mean and standard deviation for numerical variables. The Kolmogorov–Smirnov test was used to determine numerical data distribution. The Mann–Whitney U test and the independent samples t-test were used to evaluate the significance of the differences between the groups, as appropriate. The homogeneity of the variances was examined with the Levene test, and the significance level was taken as *p* < 0.05.

## Results

All the participants in each group were male. The study included 41 patients in Group 1 who met the predefined inclusion criteria and 42 patients in Group 2. The mean age of participants in Group 1 was 36.1 ± 13.3 years, and in Group 2, it was 34.9 ± 11.5 years. Statistical analysis revealed no significant difference in the age distribution between the two groups (*p* = 0.229). Baseline patient characteristics were summarized in Table 1.

**Table 1:** Demographic and baseline clinic characteristics

		Group 1 (*n*:41) Mean ± SD	Group 2 (*n*:42) Mean ± SD	*p*
Age (years)		36.1 ± 13.3	34.9 ± 11.5	0.229
Time between trauma and first visit (hours)		7.3 ± 3.4	8.2 ± 3.3	0.132
Side of trauma (R/L)		18/23	22/20	0.099
CFB size (*n*)	≤ 1 mm	19	17	0.101
	>1 mm	22	25	

R: right, L: left, CFB: corneal foreign body, SD: standard deviation

There was no statistically significant difference between the groups regarding the corneal surface annuli where the participants’ foreign bodies were located, their depth, or the size of the foreign bodies (*p* > 0.05 for all). (Tables 1 and 3)

Pre-treatment, TBUT was 10.7 ± 2.5 s in Group 1 and 11.2 ± 2.4 s in Group 2, with no significant difference between the two groups (*p* = 0.445). Similarly, mean Schirmer test results were 16.8 ± 4.7 mm and 17.1 ± 3.9 mm in Groups 1 and 2, respectively, demonstrating no statistically significant difference (*p* = 0.866). Post-epithelial healing, a comparison of dry eye tests revealed no significant changes in Schirmer test values in either group (*p* > 0.05 for both comparisons). However, TBUT demonstrated significant prolongation in both groups (*p* = 0.012 and *p* < 0.01 in Groups 1 and 2, respectively), and Group 2 demonstrated significantly higher post-treatment TBUT values compared with Group 1(*p* = 0.022), although baseline measurements were similar between the groups. There was no significant difference in baseline corneal densitometry between the two groups (*p* = 0.399). Post-epithelial healing, both groups showed a significant reduction in densitometry compared with their initial measurements (*p* < 0.05 for both). Notably, the group receiving AS treatment demonstrated a significantly lower corneal densitometry level compared with the group, which was treated without AS (*p* = 0.007). (Table 2)

**Table 2:** The changes in parameters with treatment regimens

		Group 1	Group 2	P*
Corneal density (GSU)	Pre-treatment Post-treatment	18.2 ± 2.4	17.5 ± 1.9	0.399
		15.5 ± 2.8	13.3 ± 1.2	**0.007**
UCDVA (logMAR)	Pre-treatment Post-treatment	0.201 ± 0.072	0.230 ± 0.082	0.112
		0.018 ± 0.044	0.022 ± 0.051	0.084
Tear breakup time (TBUT), s	Pre-treatment Post-treatment	10.7 ± 2.5	11.2 ± 2.4	0.445
		13.5 ± 3.1	14.9 ± 3.8	**0.022**
Schirmer test, mm	Pre-treatment Post-treatment	16.8 ± 4.7	17.1 ± 3.9	0.866
		17.2 ± 3.6	17.3 ± 3.4	0.240

GSU: Grayscale unit, UCDVA: Uncorrected distance visual acuity, TBUT: Tear breakup time, Bold, significant differences, *: Independent-Samples T-test

The initial epithelial defect size was comparable between the two groups, with mean sizes of 4.22 ± 1.61 mm² and 4.49 ± 1.57 mm² in Groups 1 and 2, respectively (*p* = 0.098). However, the mean time to complete epithelial healing was significantly shorter in Group 2 (2.19 ± 1.10 days) compared with Group 1 (3.51 ± 1.03 days) (*p* < 0.001). This finding was consistent with a significantly faster epithelial healing rate in Group 2 (*p* = 0.002). (Table 3) When both groups were further subdivided based on whether the baseline epithelial defect size was greater than 4 mm², patients in Group 2 exhibited significantly faster healing rates in both the subgroups with defects larger than 4 mm and the subgroup with defects smaller than 4 mm. (*p* < 0.05 for all). Additionally, analysis of corneal defect depth revealed that epithelial/basement membrane defects healed significantly faster in patients treated with AS (*p* = 0.003). However, no significant difference in healing time was observed between the two treatment groups for deeper lesions (*p* > 0.05 for both).

**Table 3:** Corneal parameters

		Group 1 (*n*:41) Mean ± SD	Group 2 (*n*:42) Mean ± SD	*p*
Epithelial defect size (mm^2^)		4.22 ± 1.61	4.49 ± 1.57	0.098
Epithelial healing time (days)		3.51 ± 1.03	2.19 ± 1.10	**< 0.001**
Epithelial healing rate (mm^2^/day)		1.45 ± 0.63	2.37 ± 1.54	**0.002**
Depth of defect (*n*)	E/BM	17	18	0.441
	Anterior stroma	15	14	0.847
	Mid-stroma	9	10	0.902

E: epithelium, BM: basement membrane, SD: standard deviation, Bold, significant differences

## Discussion

In this study, the standard of care, as outlined in the Wills Eye Manual, involves atraumatic rust ring removal, and antibiotic prophylaxis was applied [13]. Mechanical trauma associated with foreign body extraction often results in corneal epithelial defects. Patients are typically followed until complete re-epithelialization, which occurs through a centripetal progression [14, 15].

A significant portion of CFBs occurs because of occupational accidents and predominantly affects young male individuals. In this study, all patients were male with an average age of 36.2 years. Considering the impact on individuals in their active years and their expected lifespans, the burden of potential complications could be substantial. In our study, we demonstrated that the use of topical AS following CFB removal could have an additive effect on conventional treatments, aiming to shorten the duration of symptoms of epithelial defects and dry eye findings.

After corneal epithelial injury, the response is initiated very quickly within the first hour [16]. Some cytokine modulators act as regulators of the response [17]. Released growth factors and cytokines facilitate the organization of a new basement membrane; the corneal surface epithelium proliferates, resulting in an epithelial plug that fills the defect and completes epithelial healing [16]. EGF, TGF-β, and fibronectin present in autologous serum accelerate epithelialization [18]. Additionally, autologous tears enhance stromal corneal wound healing by increasing matrix metalloproteinase activity [19]. In this study, consistent with the literature, a significant increase in the rate of corneal defect closure following CFB was achieved with the use of topical AS.

In a study by Sul et al. involving patients who underwent pterygium surgery, those treated with AS demonstrated a 2-day earlier completion of corneal epithelialization compared with those treated with artificial tears [20]. Additionally, patients in the autologous serum group reported significantly lower postoperative pain scores during the first 5 days [20]. The accelerated re-epithelialization of the cornea may contribute to reduced ocular pain after pterygium surgery. In a study of diabetic patients undergoing combined cataract surgery and vitrectomy, where corneal epithelial debridement was performed for enhanced intraoperative visualization, patients treated with AS achieved a mean epithelialization time of 4.3 days, while those treated with hyaluronic acid had a mean time of 7.1 days, suggesting that AS may be more effective in accelerating epithelialization in the presence of comorbidities like diabetes that impair healing [21].

Autologous serum demonstrated promising results in treating patients with aqueous deficient dry eye and persistent epithelial defects [22, 23]. Freire et al. showed that not only AS but also other blood derivatives can accelerate corneal healing in both vivo and vitro models [11]. A previous study found that topical gatifloxacin and moxifloxacin can delay epithelial healing, and most topical antibiotics are cytotoxic to the corneal epithelium, potentially causing superficial punctate keratopathy [24]. Rebattu et al. reported infectious keratitis in 4.9% of patients with CFBs despite antiseptic and antibiotic treatment [4]. In our study, to reduce the risk of infectious keratitis, we added prophylactic moxifloxacin to both treatment regimens. No cases of infectious keratitis were observed in the present study.

The potential for ocular infections caused by contaminated eye drops is a significant risk associated with the use of AS, just as it is with other medications [25]. Patient education regarding appropriate storage conditions is essential to mitigate this risk. Studies indicated that the growth factors EGF, vitamin A, and TGF-β in AS remain stable for up to 1 month when refrigerated and up to 3 months when frozen [5]. A study utilizing 100% AS eye drops reported high epithelial healing rates (93.92%) and a low complication rate, suggesting that the higher growth factor concentrations and reduced manipulation associated with this approach may contribute to a lower risk of contamination [26]. Recognizing the risk of contamination inherent to AS, we provided comprehensive patient education on appropriate storage and administration techniques. Considering this risk, we suggest that the use of AS may be more advantageous in the management of acute conditions like corneal foreign body injuries, which generally necessitate shorter treatment durations, as compared with chronic ocular surface disorders such as persistent epithelial defects.

We observed that superficial epithelial defects healed significantly faster in the AS group, whereas deep defects did not show a difference between the two groups. Given that an intact basement membrane is crucial for epithelial healing and serves as a binding site for growth factors such as TGF-β, platelet-derived growth factor (PDGF), and keratinocyte growth factor (KGF) [27], the growth factors delivered by AS may have been more effective in promoting healing in the presence of a relatively intact basement membrane.

Maintaining corneal transparency would be a positive factor for visual prognosis, particularly in individuals presenting with a foreign body in the central cornea or those working without protective eyewear and experiencing repeated corneal foreign body trauma. Our study demonstrated that AS not only accelerated epithelial healing but also improved corneal transparency as evidenced by reduced densitometry values. These findings may suggest that AS may promote long-term corneal health by enhancing cellular turnover and facilitating the restoration of normal tissue architecture.

In the literature, there are studies indicating that the use of 20% AS does not affect Schirmer’s with anesthesia results in Sjögren’s patients [28], while the use of 50% AS improves Schirmer’s with anesthesia test outcomes [29]. In our study, Schirmer’s with anesthesia results in both treatment groups did not show significant changes with the closure of the epithelial defect. We attribute this to performing the Schirmer test with topical anesthesia to prevent reflex tearing, and to the lack of consideration of any potential negative impact of corneal foreign body trauma on aqueous tear production. In contrast to the Schirmer test results, TBUT values improved in both groups, with this improvement being more pronounced in the AS group. This observation may be attributed to the potential of AS to enhance corneal healing and improve tear film stability [30].

Limitations of the study include the fact that due to the unavailability of anterior segment optical coherence tomography (OCT); the depth of the defect could not be accurately measured. When patients were divided into subgroups based on defect depth, small participant subgroups were obtained, and pain scoring was not performed during visits. Patients using AS, compared with the other group, used the same amount of topical artificial tears and antibiotics but ended up using a higher total number of drops by the end of the day, demonstrating the additive effect in the treatment. Finally, patients were evaluated until the epithelial defect closed, and the medium- and long-term outcomes of AS instillation after CFB removal were not examined.

In conclusion, AS increased the rate of corneal defect closure following metallic CFB traumas. As the size of the epithelial defect increased, the effect of AS in addition to topical antibiotics and artificial tears became more pronounced. The use of AS had a positive impact on reducing increased corneal light scatter, a common sequela of CFB trauma or its removal. Contamination-controlled AS drops can aid in the closure of corneal defects in short-term treatments, as seen in metallic CFB patients, and can expedite the return to routine activities for young individuals in active working life.

## Data Availability

The data used to support the findings of this study are included in this article.
